# Effects of preventive home visits on health care costs for ambulatory frail elders: a randomized controlled trial

**DOI:** 10.1007/s40520-013-0128-4

**Published:** 2013-08-15

**Authors:** Ayumi Kono, Yukiko Kanaya, Chieko Tsumura, Laurence Z. Rubenstein

**Affiliations:** 1 School of Nursing, Osaka City University, 1-5-17 Asahi Abeno, Osaka, 545-0051 Japan; 2 School of Nursing and Rehabilitation, Konan Women’s University, Kobe, Hyogo Japan; 3 Department of Geriatric Medicine, College of Medicine, The University of Oklahoma, Oklahoma City, OK USA

**Keywords:** Frail elderly, Health care costs, Hospitalization, Home visits, Randomized controlled trial

## Abstract

**Background and aims:**

Reducing health care costs through preventive geriatric care has become a high priority in Japan. We analyzed data from a randomized controlled trial to examine the effects of a preventive home visit program on health care costs among ambulatory frail elders.

**Methods:**

Structured preventive home visits by nurses or care managers were provided to the visit group every 6 months over 2 years. The enrolled participants (*N* = 323) were randomly assigned to either the visit group (*N* = 161) or the control group (*N* = 162). We analyzed the health care costs, including the costs for hospitalizations and outpatient clinic utilization for participants who had health care insurance from the local government (*N* = 307). The visit group included 154 individuals in the visit group and 153 people in the control group.

**Results:**

Total health care costs over the study period were not significantly different between groups, but at most monthly time points costs and those for outpatient clinic utilization in the visit group were lower than those in the control group. Hospitalizations, which accounted for more than ¥500,000 JPY per month, were less likely to occur more often among participants in the visit group (*N* = 71) than in the control group (*N* = 113) (OR = 0.63; *p* = 0.002).

**Conclusions:**

These results suggest that a preventive home visit program may reduce monthly health care costs, primarily by reducing hospitalization costs.

## Introduction

Several studies have recently documented the effects of preventive home visit programs for community-dwelling older individuals [1–5]. Particularly favorable outcomes have been associated with certain population subgroups based on multidimensional assessments and multiple follow-up home visits [6–8].

The positive effects of preventive home visits have been documented with regard to functional and psychosocial parameters and health care utilization. However, the results have been inconsistent. Some studies have not conclusively found that home visit programs are cost-effective [9, 10]. Other studies have demonstrated the cost effectiveness of home visit programs in terms of a reduction in the utilization of emergency health services among elderly individuals [11–13] or a delay in their first emergency admission to a hospital [13]. A randomized controlled trial (RCT) suggested that the hospital and institutional costs for subjects receiving home visits were less than those for subjects who were not receiving home visits; however, among subjects receiving home visits, the home care, adult day care, and meals-on-wheels costs were increased, which offset much of the savings [14]. Most of these trials were conducted in European countries [9, 10, 12–14] or the United States [11], and the results have varied across national settings and systems.

Thus, there is no simple answer regarding the effects of home visits or their cost-effectiveness [6], and more evidence is required in various countries and health care settings.

Japan provides a particularly fertile ground for such studies. First, Japan has become the most aged society worldwide, with a low birth rate and high longevity, and the proportion of elderly individuals aged 65 years or older in the population reached 23.1 % in 2010 and is projected to reach 40.5 % in 2055 [15].

Second, since 2000, the Japanese government has operated a unique system of mandatory public long-term care insurance (LTCI) that is based on social insurance principles that mandate benefits regardless of income or family situation [16]. Formal facility-based care (including nursing homes, group homes, and respite care) and community-based care (including adult day care, home aid, home modifications, and partial visiting nursing care) are reimbursed by the LTCI program.

Finally, Japan provides universal health coverage through employee-based or community-based social health insurance programs [17]. In 2010, the proportion of individuals aged 60 years or older who utilized health care services more than once per week was 61.6 %. This proportion is higher than those reported in other countries, such as the United States (24.6 %), Germany (32.9 %), and Sweden (14.6 %) [15].

Japan faces a challenge inherent in social health insurance [17] or LTCI. Although improving health care by preventing geriatric syndromes and elderly functional decline has been one of the highest priorities, the increasingly aging society ultimately raises the cost of care. In the past decade, several RCTs [18–20] have examined the effects of preventive home visit programs for frail elderly Japanese people, but these studies have not presented outcomes related to health care costs.

Based on our multidimensional assessment model of preventive home visits [21], we previously reported a RCT that demonstrated that these home visits were effective for improving the functional status and depression among ambulatory frail elderly people with dependency in activities of daily living (ADLs) [22]. We hypothesized that the preventive home visit program may also be able to reduce health care costs, which is the subject of the present analysis [22]. The aim of this study was to examine the effects of a preventive home visit program on health care costs for hospitalizations and outpatient clinic utilization in ambulatory frail elderly people over a 2-year follow-up period.

## Methods

### Procedure

A single-blind randomized controlled trial was performed in 3 suburban municipalities in Osaka, Japan, and the subjects were followed for 2 years. Details on the procedures and selection of the study participants have been previously published [21, 22].

The purpose of the study and the process of analyzing the health care cost data from local government documents were explained to the study participants. The present study protocol was approved by the Nursing Research Ethical Committee of Osaka City University (No. 19-3-3, October 01, 2007) and was registered at the UMIN clinical trials registry approved by ICMJE (No. UMIN000001113, April 07, 2008).

### Participants

Operational definition of ambulatory frail elders was as being classified into the two lowest care need levels in the LTCI system: Support Levels 1 and 2 (out of 7). The participants were identified from the list of LTCI-certified residents that is maintained at each local government office. The eligibility criteria included the following: (1) age of 65 years or older, (2) certified as Support Level 1 or 2 in the LTCI system, (3) living at home at the time of the baseline survey, and (4) not having utilized formal long-term care services that are reimbursed by the LTCI system in the previous 3 months. We focused on eligible subjects without recent utilization to examine the effects of the program on this unique subgroup and the ability of the program to prevent the utilization of long-term care services [22].

There were 1,764 elders who were certified as Support Level 1 or 2 at the end of September (1 municipality) and November (2 municipalities) of 2007. Of these individuals, 568 were eligible (i.e., not using long-term care services) to participate in the baseline survey, which was conducted between December 2007 and February 2008. After the baseline survey, 323 participants remained eligible and were willing to be randomly assigned to either the visit group (*N* = 161) or the usual care group (*N* = 162) by researchers using computer-generated random numbers stratified by sex, age group, and district within each community.

The characteristics of the original study participants at baseline are shown in Table 1: their mean age was 80 years, 74 % were females, and their mean ADL scores measured on the Barthel Index [23] were approximately 90 out of 100.

**Table 1:** Characteristics of the original study participants at baseline (*N* = 323)

	Group	
	Intervention *N* = 161	Control *N* = 162
Age	80.3 (6.7)	79.6 (6.4)
Female, *N* (%)	119 (73.9)	120 (74.1)
Support Level 1 (lowest LTCI certification level), *N* (%)	76 (47.2)	79 (48.8)
Living alone, *N* (%)	43 (26.7)	47 (29.0)
ADLs^a^, Mean (SD)	90.2 (11.7)	91.4 (12.2)
Type of health care insurance		
Prefecture-level health insurance of elders aged at least 74 years, *N* (%)	130 (80.8)	127 (78.4)
Municipality-level health insurance, *N* (%)	24 (14.9)	26 (16.0)
Employee-based health insurance, *N* (%)	4 (2.5)	5 (3.1)
Public assistance, *N* (%)	3 (1.9)	4 (2.5)

^a^ADLs were measured using the Barthel Index. The scores ranged from 0, which represents an unfavorable ADL, to 100, which represents a favorable ADL

The types of health insurance used by the participants are also shown in Table 1: “Prefecture-level health insurance” covers elderly individuals aged 75 years or older, and “municipality-level (city or town-level) health insurance” covers individuals aged 74 years or younger. Typically, Japanese individuals have community-based health insurance after retirement, although some individuals continue to have health insurance from their former employers even after retirement. Individuals who receive public assistance are not enrolled in any social health insurance, and can receive health care without charge in any hospital or clinic [17].

A total of 307 participants in the present analysis were elderly individuals who had community-based health insurance (at the prefecture or municipality level), with 154 people in the visit group (mean age 80.5 ± 6.3 years; 72.7 % female) and 153 people in the control group (mean age 79.9 ± 6.6 years; 73.9 % females).

### Data collection

The monthly health care costs for all types of hospitalization, including acute care, mental health care, tuberculosis care, and rehabilitation care, as well as outpatient clinic utilization were included in the analysis. Over the 2-year study period, records from both prefecture- and municipality-level health insurance plans were collected from local government offices.

### Preventive home visits

For elders in the visit group, routine preventive home visits were conducted every 6 months for 2 years by community health nurses or care managers who were affiliated with municipal community-based comprehensive care centers (according to the LTCI reform plan of 2005). The visits included structured multidimensional interview-based assessments of five key elements: locomotion, daily activities, social contacts or relationships with other people, health conditions, and signs of abuse [21]. Home visitors documented 40 potential health or psychosocial problems or difficulties and provided recommendations to each individual elder or caregiver [21]. All four visits were completed for the majority of participants (1st visit 87 %, 2nd visit 85.7 %, 3rd visit 83.9 %, and 4th visit 83.9 %). Further information can be found in our previous articles [21, 22]. We paid ¥5,000 JPY (approximately $50 USD) per person per year for the present preventive home visits from our research grant to municipal community-based comprehensive care centers.

### Statistical analysis

All analyses were conducted on an intention-to-treat basis (including cases in which participants refused the intervention after the randomization). The software program SAS version 9.2 was used, and a 2-tailed probability level of less than 0.05 indicated statistical significance. At the time of the 2-year follow-up, 11 individuals in the visit group and 20 individuals in the control group had died, and 5 individuals in the visit group and 3 individuals in the control group had been institutionalized. We included the data from the participants who had died, had been admitted to the hospital, or had been institutionalized during the study period. However, data from participants who had moved out of the study area (six individuals in the visit group and six individuals in the control group) were not included, as follow-ups were not possible.

Because the kurtosis and skewness of the health care cost values were high, the log-transformed values of the total or monthly health care costs and health care costs for outpatient clinic utilization were evaluated using a *t* test.

Every month during the study period the number of participants who were hospitalized ranged from 5 to 15, and the individual raw data of the monthly hospital health care costs were plotted to compare the distribution between the groups. The monthly hospital care refers to the continuous cost for hospitalization within a month, and we did not count the number of hospitalizations or hospitals. For example, for a person hospitalized from January 1 to February 10 at a hospital, we counted the costs from January 1 to January 31 and from February 1 to February 10 separately to provide the costs of January and February, respectively. The difference in the number of hospitalizations that accounted for more than ¥500,000 JPY per month per month was examined between the groups according to Fisher’s exact test because mean health care cost for a hospitalization (Mean of hospital stay days was 17.1 days) on elderly people aged 70 years or older in 2010 was ¥502,708 JPY [24].

## Results

### Total health care costs between groups

The mean health care costs over the 2-year period were slightly lower in the visit group (mean ± SEM ¥2,016,606 ± 161,432 JPY, approximately $20,166 ± 1,614 USD) than in the control group (¥2,287,450 ± 200,535 JPY, approximately $22,875 ± 2,005 USD), but the difference was not significant (difference of 95 % CI = −0.113 to 0.294; *p* = 0.38) when log-transformed values were used for the *t* test.

**Fig. 1:**
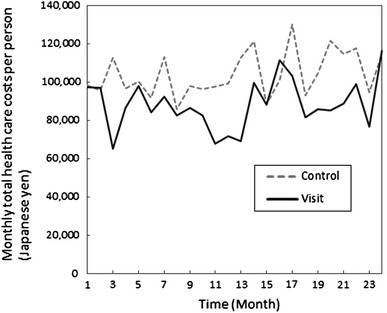
Monthly total health care costs of the groups during the study period (the visit group *N* = 154; the control group *N* = 153). (**a**) Numbers are raw data. (**b**) The yearly TTS average for 2008 was 1 USD = 104.5 JPY; this average for 2009 was 1 USD = 94.6 JPY. (**c**) A* t* test showed significant differences of each health care cost between groups using log-transformed values at 3M (*p* = 0.03), 11M (*p* = 0.005), 12M (*p* = 0.046), 13M (*p* = 0.01) and 17M (*p* = 0.049)

The changes in total health care costs per month throughout the study period between the two groups are shown in Fig. 1. At most monthly time points, the costs in the visit group were lower than those in the control group, and the costs in the visit group were significantly lower than those in the control group at 3 months (difference of 95 % CI = 0.017–0.489; *p* = 0.03), 11 months (0.102–0.566; 0.005), 12 months (0.003–0.434; 0.046), 13 months (0.051–0.542; 0.01), and 17 months (0.0004–0.534; 0.049) as determined using log-transformed values according to the *t* test.

### Health care costs for outpatient clinic utilization between groups

In the visit group, the mean per-person health care cost for outpatient clinic utilization over the 2-year period was ¥1,337,246 ± 102,913 JPY (approximately $13,372 ± 1,029 USD), which was lower than the mean cost in the control group (¥1,637,667 ± 148,826 JPY, approximately $16,377 ± 1,488 USD). However, this difference did not quite reach statistical significance (difference of 95 % CI = −0.003 to 0.355; *p* = 0.053) when log-transformed values were used.

**Fig. 2:**
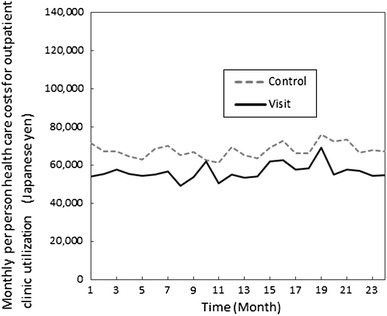
Monthly health care costs for outpatient clinic utilization of the groups during the study period (the visit group *N* = 154; the control group *N* = 153). (**a**) Numbers are raw data. (**b**) The yearly TTS average for 2008 was 1 USD = 104.5 JPY; this average for 2009 was 1 USD = 94.6 JPY. (**c**) A* t* test showed significant differences of each health care cost between groups using log-transformed values at 1M (*p* = 0.01), 6M (*p* = 0.01), 7M (*p* = 0.04), 8M (*p* = 0.008), 11M (*p* = 0.02), 12M (*p* = 0.02), 21M (*p* = 0.01) and 23M (*p* = 0.048)

The per-person changes in the monthly health care costs of outpatient clinic utilization are shown in Fig. 2. These costs were lower in the visit group than in the control group at all monthly time points, and the costs in the visit group were significantly lower than the costs in the control group at 1 month (difference of 95 % CI = 0.065–0.493; *p* = 0.01), 6 months (0.057–0.455; 0.01), 7 months (0.008–0.377; 0.04), 8 months (0.075–0.492; 0.008), 11 months (0.034–0.448; 0.02), 12 months (0.034–0.409; 0.02), 21 months (0.055–0.501; 0.01), and 23 months (0.001–0.435; 0.048) as determined using log-transformed values according to the *t* test.

### Hospital care costs per person per month between groups

The mean hospital care costs over the 2-year period in both groups were almost same, mean visit group cost was ¥679,359 ± 119,827 JPY (approximately $6,794USD), and mean control group cost ¥639,484 ± 124,511 JPY (approximately $6,395 USD) This difference was not significant (difference of 95 % CI = −0.036 to 0.355; *p* = 0.49) using log-transformed values for the *t* test.

**Fig. 3:**
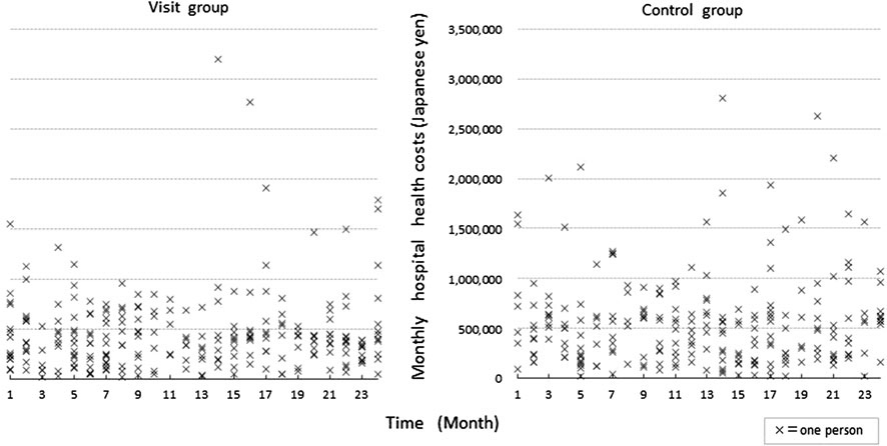
Distribution of monthly hospital care costs of the groups during the study period (*N* = 307). (**a**) The yearly TTS average for 2008 was 1 USD = 104.5 JPY; this average for 2009 was 1 USD = 94.6 JPY. (**b**) The number of participants who were hospitalized in the visit group were ordered from 1 to 24 months;* N* = 14, 13, 6, 10, 15, 14, 15, 11, 11, 7, 5, 7, 8, 9, 10, 11, 10, 8, 7, 9, 10, 13, 11, 13. (**c**) The number of participants who were hospitalized in the control group were ordered from 1 to 24 months;* N* = 6, 10, 9, 9, 14, 7, 10, 5, 9, 10, 11, 9, 10, 13, 9, 11, 14, 9, 6, 9, 10, 10, 6, 10. (**d**) Hospitalizations that accounted for more than 500,000 JPY: the visit vs. control group (*N* = 71 vs.* N* = 113); OR = 0.63,* p* = 0.002

The distribution of monthly hospital care costs between the groups is shown in Fig. 3. The number of participants who were hospitalized per month ranged from 5 to 15 in the visit group and from 5 to 14 in the control group.

Over the 2-year study period, the more costly hospitalizations, which were defined as those costing more than the mean of ¥500,000 JPY (approximately $5,000 USD) per person per month, occurred significantly more often (OR = 0.63; 95 % CI = 0.46–0.84; *p* = 0.002) in the control group (*N* = 113) than in the visit group (*N* = 71).

## Discussion

Total health care costs over the study period were not significantly different between groups; but at most monthly time points, total costs and those for outpatient clinic utilization were lower in the visit group than those in the control group. Moreover, hospitalizations that cost more than ¥500,000 JPY per month were less likely to occur among participants in the home visit group than among participants in the control group.

The present analysis has shown that the preventive home visit program, which consists of structured multidimensional assessments and recommendations from community health nurses and care managers, may reduce monthly health care costs for ambulatory frail elderly individuals who are certified as needing long-term care services, particularly avoiding costly hospitalizations.

We previously reported that the current preventive home visit program facilitates the earlier use of public long-term care services among ambulatory frail elderly individuals [22]. These findings are similar to those of another report [14] that found lower hospital and institutionalization costs but higher home care and adult day care costs associated with a preventive home visit program. Our interpretation of these results is that the recommendations of home visitors increased the focus on the use of long-term care services [22], which tends to prevent costly hospitalizations. Community health nurses or care managers who visited the study participants were likely able to assess the risks of serious health conditions and provide preventive recommendations, including the use of long-term care services. The use of long-term care services, especially home-based long-term care services, may be effective in preventing serious health conditions because long-term service providers can detect health changes early.

Moreover, the long-term care costs over the study period, which were ¥378,010 JPY (approximately $3,780 USD) in the visit group and ¥273,231 JPY (approximately $2,732 USD) in the control group [22], only amounted to approximately 10 % of the total health care costs over the same 2-year period (visit group: ¥2,016,606 JPY, approximately $20,166 USD; control group: ¥2,287,450 JPY, approximately $22,875 USD). Since remaining independent in the home is valuable for older people, even if it requires the use of in-home long-term care services, cost-containment approaches should focus on reducing the total health care costs that are incurred by illness or disability (e.g., through the preventive home visit approach) rather than simply reducing the long-term care costs alone.

The Statistical Bureau of the Japanese Ministry of Internal Affairs and Communication reported that the average annual per-person health care expenditures for the Japanese population aged 75 years and older were ¥865,146 JPY (approximately $8,651 USD) in 2008 and ¥882,118 JPY (approximately $8,821 USD) in 2009 [25]. Thus, the total national average health care expenditure over our 2-year study period for each Japanese elder (2008–2009) was approximately ¥1,747,264 JPY (approximately $17,472 USD). This amount is lower than the expenditures for both groups in our analysis (visit group: ¥2,016,606 JPY, approximately $20,166 USD; control group: 2,287,450 JPY, approximately $22,875 USD), which is consistent with our study participants being frailer and older and requiring more medical care than the general elderly population. In Japan, the 2009 life expectancy at birth was 83 years [26], which is the longest life expectancy in the world; in 2009, health care expenditures comprised 9.5 % of the nation’s gross domestic product, which is lower than that in many other countries [26], and the Japanese health care system seems to work well [27]. However, as mentioned previously [15], the proportion of elders in the Japanese population will soon reach more than 40 %, and the number of frail elderly individuals is increasing. These changes are associated with rising health care expenditures for the types of frail elderly people who comprised our study population, which creates a major concern with regard to health care financing [28].

Even though health care costs and those for outpatient clinic utilization in the visit group were lower than those in the control group at most monthly time points of the period, the differences in total health care costs, those for outpatient clinic utilization or those for hospitalization over the period were not statistically significant between groups. This is likely because preventive home visit programs do not show their full effects immediately.

There are several limitations in the present study. First of all, a limitation of the present study is that we did not have detailed data regarding the exact medical diagnoses and treatments that were reimbursed by health care insurance programs in the outpatient clinics or hospitals for the study participants. In particular, we were unable to evaluate the processes of care or the reasons why the number of costly hospitalizations was reduced in the visit group relative to the control group. Moreover, the average hospital stay in Japanese elders aged 65 years or older is 44 days [29]. However, some of hospitalizations may have been counted as less than ¥500,000 JPY in the present study, though they were part of consecutive hospitalizations that actually cost more than ¥500,000 JPY.

Second, we were unable to analyze the costs for the small number of participants who had employee-based health insurance or public assistance. The number of older individuals in Japanese metropolitan areas who depend on public assistance is currently increasing [17], and they tend to consume a disproportionate amount of medical resources [30] even though they do not represent the majority of the elderly population.

Third, our statistical analysis for testing differences of health care costs at each monthly time point may have overestimated statistical significance due to multiple comparisons. In the future, a more rigorous and detailed statistical analysis should be conducted for examining health care costs of preventive home visits.

A further limitation of our study is that our preventive home visit program is currently adapted to a local government population-based approach rather than a primary care system. Further preventive home visit programs should be tested using elderly medical checkup data assessments or primary care physician assessments.

## Conclusions

The present second analysis of randomized controlled trial showed that a preventive home visit program can reduce health care costs, primarily from reduced hospitalizations, in addition to providing other major benefits. Further research should be directed toward investigating health care process during preventive home visit. Moreover, we need to develop preventive home visit programs that link better to primary care for ambulatory frail elders living at home.
